# Tocilizumab Induced Acquired Factor XIII Deficiency in Patients with Rheumatoid Arthritis

**DOI:** 10.1371/journal.pone.0069944

**Published:** 2013-08-01

**Authors:** Sho Mokuda, Yosuke Murata, Naoya Sawada, Kenichiro Matoba, Akihiro Yamada, Makoto Onishi, Yasuaki Okuda, Kazuo Jouyama, Eiji Sugiyama, Kiyoshi Takasugi

**Affiliations:** 1 Department of Clinical Immunology and Rheumatology, Hiroshima University Hospital, Hiroshima City, Hiroshima, Japan; 2 Department of Internal Medicine, Center for Rheumatic Diseases, Dohgo Spa Hospital, Matsuyama City, Ehime, Japan; INSERM-Université Paris-Sud, France

## Abstract

Factor XIII is one of the twelve coagulation factors and also known as a fibrin-stabilizing factor. In 2012, we encountered a male RA patient with hemorrhagic factor XIII deficiency who had been treated with tocilizumab for two years. There are few reports regarding the relationship between tocilizumab (a humanized monoclonal antibody against the interleukin-6 receptor (IL-6R)) and factor XIII. We measured the factor XIII activity levels in the plasma of 40 RA patients (10 patients treated without biologics, 30 patients treated with biologics (15 patients treated with necrosis factor inhibitors and 15 patients treated with tocilizumab)) and 19 healthy controls. Consequently, the tocilizumab group exhibited lower levels than the other three groups according to the Steel-Dwass test (P<0.01). Furthermore, we compared the plasma factor XIII activity levels and the plasma factor XIII concentrations in the RA patients treated with biologics. Pearson's correlation test was used to assess the relationship between the factor XIII activity levels and the plasma factor XIII concentrations (r = 0.449, *P* = 0.019). According to the multiple regression analysis, the treatment with tocilizumab is an independent risk factor for plasma factor XIII reduction in RA patients. In conclusion, RA patients treated with tocilizumab, an IL-6R blocker, are at risk of developing acquired factor XIII deficiency. The mechanisms underlying the reduced factor XIII activity observed in RA patients treated with tocilizumab may result from the quantitative reduction in the plasma. These data imply that IL-6 plays an important role in maintaining the factor XIII activity level.

## Introduction

Factor XIII is one of the twelve coagulation factors and also known as a fibrin-stabilizing factor. Factor XIII circulates in the plasma in tetrameric form (FXIII-A_2_B_2_) and consists of two catalytic A subunits (FXIII-A) and two carrier/inhibitory B subunits (FXIII-B) [1]–[5]. FXIII-A is a protransglutaminase and exhibits potential enzymatic activity. The FXIII-A dimer is also present in the cytoplasm of several cells, including platelets, megakaryocytes and monocytes/macrophages [6]–[8]. The concentration of FXIII-A is 100-fold higher in the cytoplasm of platelets than in the plasma. Both platelets and monocytes lack FXIII-B in their cytoplasm. On the other hand, FXIII-B is a glycoprotein that is secreted by hepatocytes [9].

Factor XIII participates in hemostasis, wound healing and the maintenance of pregnancy, and patients with congenital factor XIII deficiency suffer from bleeding tendencies, abnormal wound healing and recurrent miscarriages [10]. Factor XIII deficiency is classified as being either congenital or acquired. Most cases of congenital factor XIII deficiency are caused by defects in the FXIII-A gene. Only a few patients with FXIII-B deficiency have been identified in Japan, Italy and Germany [11]–[13]. Acquired factor XIII deficiency is frequently caused by secondary reductions in the level of factor XIII resulting from decrease due to hyposynthesis and/or hyperconsumption due to surgery or primary diseases such as leukemia, myelodysplastic syndrome, liver disease, disseminated intravascular coagulation (DIC), Henoch-Schoenlein purpura, etc. [10]. Only a small number of hemorrhagic cases caused by anti-factor XIII inhibitors have been reported.

Tocilizumab, a biologics and immunosuppressant, is a humanized monoclonal antibody against the interleukin-6 receptor (IL-6R). Treatment with tocilizumab blocks interkeukin-6 (IL-6) signaling within inflammatory and immune cells, and improves the clinical symptoms of autoimmune diseases, such as rheumatoid arthritis (RA), juvenile idiopathic arthritis (JIA) and multicentric Castleman's disease (MCD) [14].

In 2012, we experienced a case of pelvic bleeding in a male patient with RA who had been treated with tocilizumab for more than two years. There are few reports regarding the relationship between tocilizumab and factor XIII. Therefore, we measured the factor XIII levels in RA patients and found lower plasma factor XIII levels among RA patients treated with tocilizumab. These data imply that IL-6 plays an important role in maintaining the factor XIII activity level.

## Materials and Methods

### Patients

We conducted a single-institute-based observational study of RA patients treated at Dohgo Spa Hospital between November 2012 and March 2013. This study was conducted at Dohgo Spa Hospital and approved by the clinical ethics committee of that institution. All of the RA patients fulfilled the ACR criteria for classification of the disease [15]. A total of 40 RA patients who had undergone medical examinations at Dohgo Spa Hospital and 19 healthy controls provided their informed consent, signed a written consent form, and contributed plasma samples for the study. RA patients with the following background factors were excluded: (a) complications of liver cirrhosis, leukemia or disseminated intravascular coagulation (DIC), (b) patients who had been treated with biologics for less than six months (when the patients were treated with biologics during measurement of the factor XIII activity), (c) patients treated with more than two biologics simultaneously and (d) patients who had undergone surgery within the previous two months. The following information was obtained from each patient's medical records: age, sex, disease history, retrospective treatment history, disease activity and disease stage. The RA disease activity was calculated as the DAS28-CRP according to the formula on the DAS (Disease Activity Score) web site [16].

### Measurement of the plasma factor XIII activity levels and plasma factor XIII quantitative concentrations

We measured the factor XIII activity levels using the Berichrom FXIII kit (Siemens, Osaka, Japan) [17]. In addition, we performed a FXIII quantitative examination using the Human Factor XIII ELISA Kit (Assaypro, Saint Charles, USA).

### Statistical analysis

The significance of the differences between two groups was determined using the Mann-Whitney U test or Student's *t*-test. When we used Student's *t* test, the normality of the data was assessed according to the chi-square test for goodness of fit and the homogeneity of the variance was assessed using Bartlett's test. The differences among three groups were estimated using the Kruskal-Wallis test, followed by the Steel-Dwass test. Contingency table analyses were performed using the chi-square test. In order to evaluate the relationship between the plasma factor XIII activity levels and the quantitative concentrations, Pearson's correlation coefficient was calculated, after which the normality of the data was assessed using the chi-square test for goodness of fit and the homogeneity of the variance was assessed using Bartlett's test. In order to evaluate the relationships between the plasma factor XIII activity levels and the other factors, Spearman's rank correlation coefficients and an adjusted multivariable analysis were used. Multivariable models were constructed using a multiple regression analysis, of the variables that remained significant at the *P*<0.10 level. The data processing and analyses were performed using the Microsoft Excel software program.

## Results

### Case report

**Figure 1:**
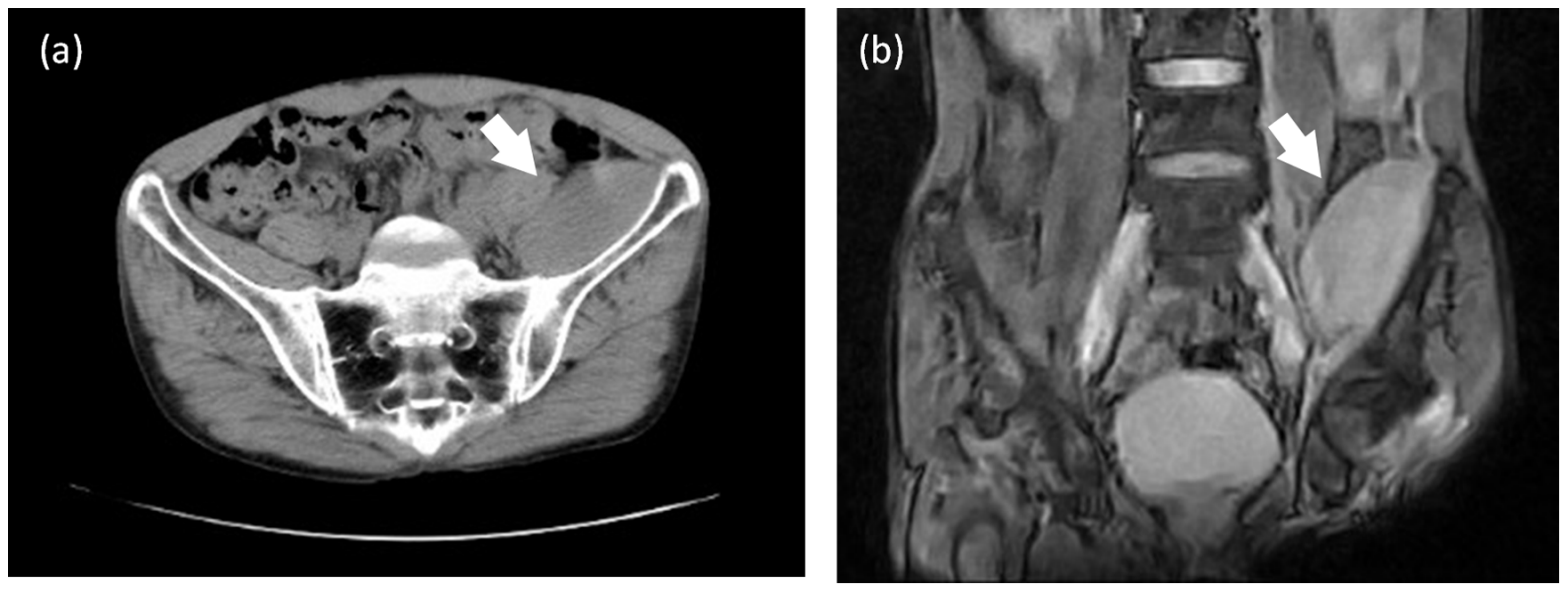
Pelvic computed tomography of the RA patient with factor XIII deficiency. The white arrow indicates the intra-pelvic hemorrhage. (a) Transverse plane and (b) coronal plane.

In May 2012, a 40-year-old male with rheumatoid arthritis (RA) presented at Dohgo Spa Hospital complaining of pain and purpura in the left inguinal region. He had been receiving treatment with tocilizumab (a monoclonal antibody against IL-6R) for two years. There were no bleeding tendencies in his family history. He had not experienced any episodes of trauma or contusion and was not suffering from hepatic cirrhosis, leukemia, DIC or vasculitis (Henoch-Schoenlein purpura). According to the blood tests, the patient's platelet count, APTT (activated partial thromboplastin time), PT (prothrombin time) and bleeding time were within the normal ranges; however, his plasma factor XIII activity level was only 58% (normal range 70–140%) (Table 1). A computed tomography (CT) scan of the pelvis revealed the presence of a mass in the left iliac muscle (Fig. 1a–b). Needle aspiration of the mass was performed, and bloody fluid was obtained. The bacterial culture was negative. A diagnosis of intra-pelvic hematoma was made. However, it was unclear why the plasma factor XIII activity level had decreased. Therefore, we researched the connection between the factor XIII activity level and rheumatoid arthritis.

**Table 1:** Coagulation tests in the RA patient with factor XIII deficiency.

Coagulation tests	(case)	standard value
Platelet	18.7×10^4^/µL	15–40×10^4^/µL
Bleeding time	60 second	60–180 second
Fibrinogen	153 mg/dl*	170–410 mg/dl
APTT	29.4 second	28–37 second
PT	11.9 second	10–13 second
FDP	1.2 µg/ml	<10 µg/ml
D-dimer	0.6 µg/ml	<1.0 µg/ml
Factor VIII activity	77 %	60–150 %
Factor IX activity	71 %	70–130 %
Factor XIII activity	58 %*	70–140 %
Factor VIII inhibitor	not detectable	not detectable
Factor IX inhibitor	not detectable	not detectable

* The data were below than the lower limit of the standard value.

APTT, activated partial thromboplastin time; PT, prothrombin time; FDP, fibrin/brinogen degradation products.

### Tocilizumab decreases the factor XIII activity levels in RA patients

We measured the factor XIII activity levels in the plasma of 40 RA patients and 19 healthy controls. The 40 RA patients comprised the following three groups: 10 RA patients treated with methotrexate (MTX) without biologics, 15 RA patients treated with TNF (tumor necrosis factor) inhibitors and 15 RA patients treated with tocilizumab. The TNF inhibitors included infliximab (nine cases), etanercept (three cases) and adalimumab (three cases). Table 2 shows the baseline characteristics of the RA patients. The median age was 60, 63 and 53 years in the three groups, respectively. The median duration of RA was 15.0, 14.0 and 14.0 years in the three groups, respectively. There were no statistically significant differences among the groups with respect to the following factors: age, sex, duration of RA, history of treatment with biologics, Steinbrocker stage, Steinbrocker class, dose of glucocorticoids and concomitant use and dose of MTX. The disease activity levels in the RA treated without biologics were higher than those observed in the patients treated with tocilizumab (*P*<0.05). The proportion of RA patients receiving concomitant MTX among those treated with TNF inhibitors was higher than that observed among those treated with tocilizumab (*P*<0.05).

**Table 2:** Comparison of the baseline RA patient characteristics.

	treated with MTX (without biologics) (n = 10)	treated with TNF inhibitors (n = 15)	treated with Tocilizumab (n = 15)	*P*-value
Age (year)	60 (49–63)	63 (49–67)	53 (45–64)	0.634 a
Sex ( Female/Male )	F 8/M 2	F 12/M 3	F 12/M 3	1.000 b
Duration of RA (year)	15.0 (10.1–32.3)	14.0 (6.8–19.5)	14.0 (8.5–23.5)	0.772 a
History of current biologics (TNFi or TCZ) (year)	–	3.0 (2.3–5.3)	3.2 (1.7–3.9)	0.221 c
Steinbrocker stage I	2 (20.0%)	1(6.7%)	0 (0%)	0.468 b
II	2 (20.0%)	4(26.7%)	4 (26.7%)	
III	1 (10.0%)	5 (33.3%)	3 (20.0%)	
IV	5 (50.0%)	5 (33.3%)	8 (53.3%)	
Steinbrocker class 1	4 (40.0%)	7 (46.7%)	9 (60.0%)	0.370 b
2	2 (20.0%)	6 (40.0%)	5 (33.3%)	
3	3 (30.0%)	2 (13.3%)	1 (6.7%)	
4	1 (10.0%)	0 (0%)	0 (0%)	
DAS28-CRP	2.16 (2.05–2.95)	1.64 (1.4–2.22)	1.46 (1.33–2.14)	0.025 * a
Dose of oral glucocorticoids (prednisolone) (mg/day)	3.5 (0–5.0)	2.0 (0.0–3.5)	2.0 (0.5–2.0)	0.735 a
Concomitant methotrexate, n (%)	–	14 (93.3%)	9 (60.0%)	0.031 ** c
Dose of methotrexate (mg/week)	8.0 (4.5–11)	8.0 (6.0–8.0)	8.0 (6.0–8.0)	0.985 a
Factor XIII activity (%)	93 (79–111)	94 (87–107)	64 (61–69)	0.0001 *** a
Factor XIII quantitative concentration (µg/ml)	–	41.5 (37.8–62.0)	25.5 (21.0–40.0)	0.039 ** c
		(n = 13)	(n = 14)	

Median (interquartile range (IQR)).

a The Kruskal-Wallis test.

b The chi-square test.

c The Mann-Whitney U test.

* Statistical significance (*P*<0.05) was determined using the Kruskal-Wallis test for differences among the three groups. There was a significant difference between the patients treated without biologics and those treated with tocilizumab (*P*<0.05), according to the Steel-Dwass method.

** Statistical significance (*P*<0.05) was calculated using the Mann-Whitney U test.

*** Statistical significance (*P*<0.01) was calculated using Kruskal-Wallis test for differences among the three groups. There were significant differences between the patients treated without biologics and those treated with tocilizumab (*P*<0.01) and between the patients treated with TNF inhibitors and those treated with tocilizumab (*P*<0.01), according to the Steel-Dwass method.

MTX, methotrexate; TNF, tumor necrosis factor; RA, rheumatoid arthritis.

**Figure 2:**
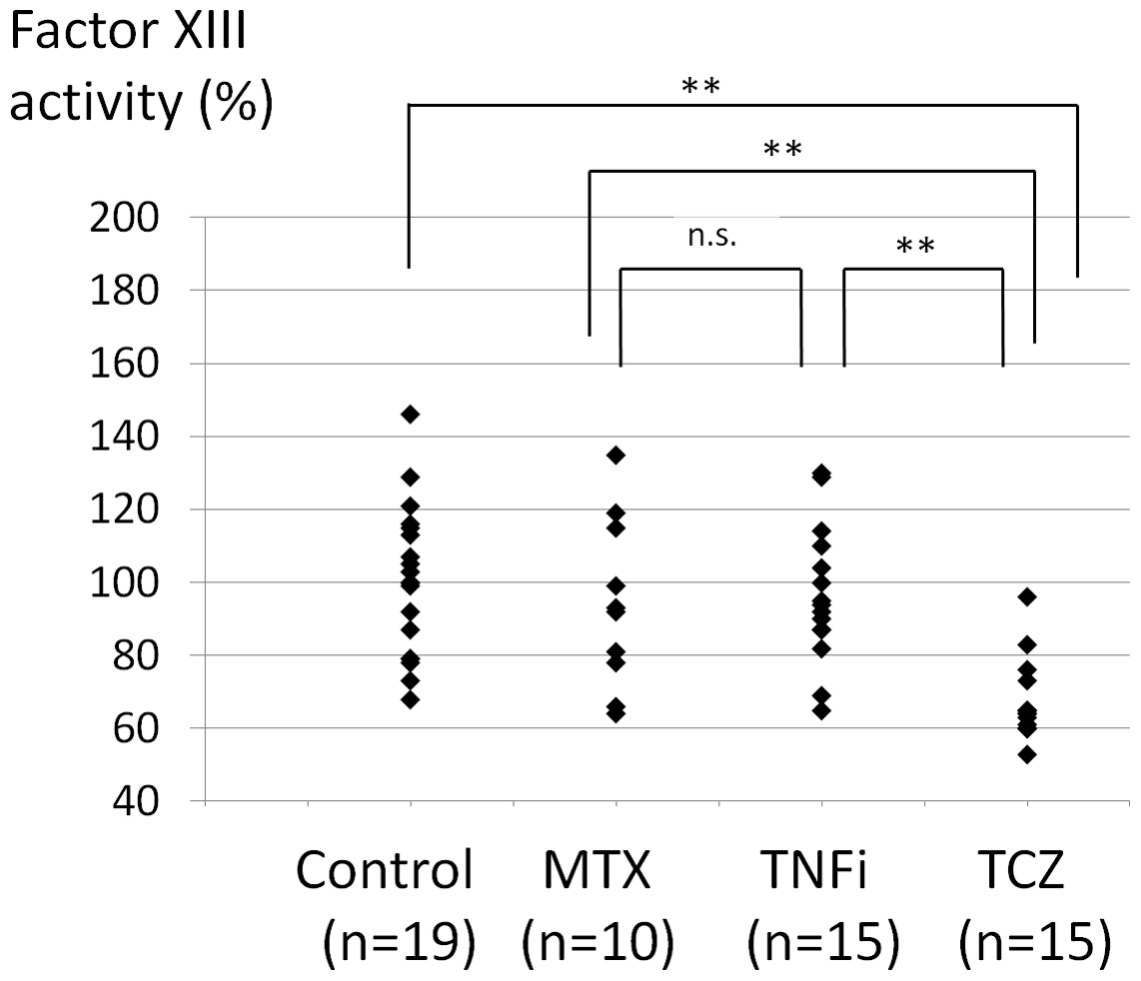
Comparison of the plasma factor XIII activity levels in the healthy controls and RA patients. ** the Steel-Dwass method (*P*<0.01). MTX, methotrexate; TNFi, tumor necrosis factor inhibitors; TCZ, tocilizumab.

As shown in Figure 2, the tocilizumab group exhibited lower levels than the other three groups (*P*<0.01). Moreover, 73% cases of the RA patients treated with tocilizumab had factor XIII activity levels below the lower limit (70%).

### The plasma factor XIII activity level is relative to the plasma factor XIII quantitative concentration in RA patients treated with biologics

**Figure 3:**
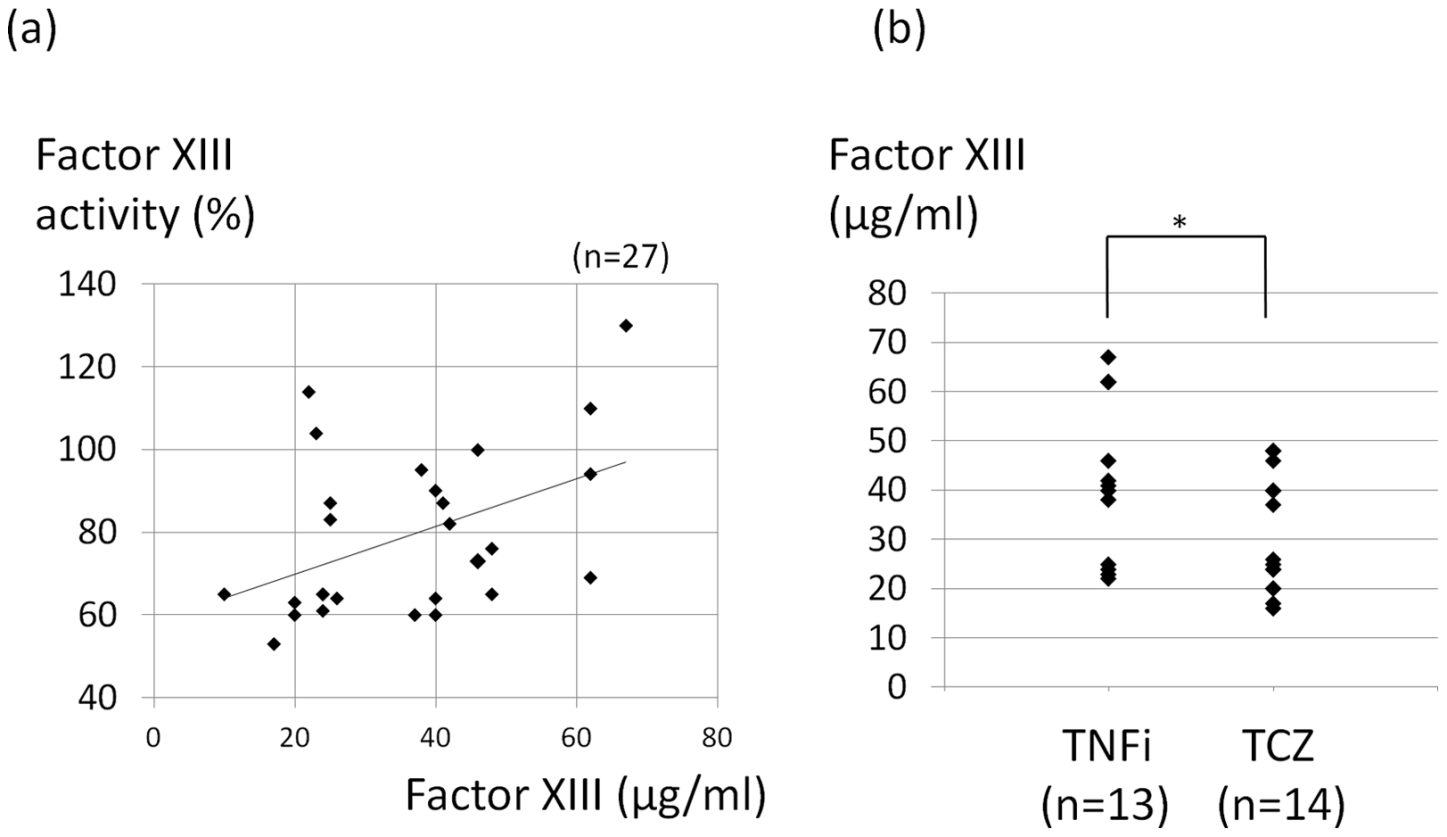
Plasma factor XIII quantitative concentrations in the RA patients treated with biologics. (a) The relationship between the factor XIII activity levels and plasma concentrations was assessed according to Pearson's correlation coefficient. (r = 0.449, *P* = 0.019). (b) Comparison of the plasma factor XIII concentrations between the RA patients treated with TNFi and those treated with tocilizumab. * Student's *t*-test (*P*<0.05). TNFi, tumor necrosis factor inhibitors; TCZ, tocilizumab.

Subsequently, we measured the plasma factor XIII quantitative concentrations in order to investigate the pathophysiological mechanisms underlying the depressed factor XIII activity induced by tocilizumab. In the RA patients treated with biologics, the plasma factor XIII concentrations were determined using ELISA. Pearson's correlation coefficient was used to assess the relationship between the factor XIII activity levels and plasma concentrations (r = 0.449, *P* = 0.019) (Fig. 3a), which revealed a positive correlation. Moreover, there was a statistically significant difference in the factor XIII concentrations between the RA patients treated with TNF inhibitors and those treated with tocilizumab (*P* = 0.041) (Fig. 3b). The origin of the factor XIII activity depression observed in patients treated with tocilizumab may be quantity factor XIII deficiency.

### Treatment with tocilizumab and a male gender are independent risk factors for the development of acquired factor XIII deficiency in RA patients

Established RA patients, often receive treatments with several immunosuppressants. We speculated whether there is a relationship between the factor XIII activity level and the use of other anti-rheumatic drugs, such as prednisolone or methotrexate. In order to evaluate the relationships between the plasma factor XIII activity levels and the other factors evaluated in this study, we used Spearman's rank correlation coefficients and an adjusted multivariable analysis. In the simple regression analyses, there were no statistically significant relationships between the factor XIII activity levels and the following factors in the 40 RA patients: age, duration of RA, history of treatment with biologics, Steinbrocker stage and class, DAS28-CRP, dose of glucocorticoids or the concomitant use or dose of MTX. The factor XIII activity levels were inversely correlated with the current use of tocilizumab (*P*<0.0001) and positively correlated with a female gender (*P* = 0.065) (Table 3). Furthermore, we performed a multiple regression analysis of the data of the 40 RA patients. The independent variables, sex and the current use of tocilizumab, exhibited correlations at the *P*<0.10 level, according to the Spearman's rank correlation coefficients, were correlated with the factor XIII activity levels in the multiple regression analysis (*P* = 0.026, <0.001, respectively) (Table 4). These findings imply that the use of tocilizumab and a male gender were independent risk factors of the factor XIII deficiency in RA patients. This phenomenon was not observed in RA patients with TNF inhibitors. When the interleukin-6 receptor is knocked down, the production or function of factor XIII may be suppressed.

**Table 3:** Single regression analyses between the factor XIII activity levels and the independent variables.

	rs	*P*-value
Age	−0.018	0.902
Sex (Male = 1, Female = 2)	0.465	0.065 *
Duration of RA	−0.096	0.541
Current use of tocilizumab	−0.421	<0.0001 ***
History of current biologics	−0.061	0.655
Steinbrocker stage	−0.129	0.209
Steinbrocker class	0.051	0.834
DAS28-CRP	0.137	0.396
Oral glucocorticoids (prednisolone) (mg/day)	−0.076	0.517
Current use of methotrexate	0.429	0.170
Methotrexate (mg/week) d	0.179	0.331

Spearman's rank correlation coefficients between the factor XIII activity levels and the other variables.

d We inserted “0” as a parameters for the patients treated without methotrexate.

* P<0.10, ***P<0.01.

**Table 4:** The multiple regression analysis of the independent risk factors for the factor XIII activity level depression.

	regression coefficient	95% CI	standard error	*P*-value
Sex (Male = 1, Female = 2)	15.1	(1.90 to 28.3)	6.51	0.026 **
Current use of tocilizumab	−28.4	(−39.3 to −17.5)	5.38	<0.001 ***

The results of the multiple regression analysis of the factor XIII activity levels, sex and use of tocilizumab. **P<0.05, ***P<0.01.

## Discussion

In 2012, we experienced a 40-year-old male RA patient treated at Dohgo Spa Hospital who suffered from intra-pelvic hemorrhage without a history episode of trauma or contusion. His plasma factor XIII activity level was 58%. After six months of recovery, the factor XIII activity level continued to be slightly low (64%). This case prompted us to wonder whether the factor XIII activity level is decreased by tocilizumab treatment. However, there are no reports showing that tocilizumab depresses the plasma factor XIII activity level. In this case, the patients' plasma fibrinogen level was also below the lower limit (Table 1). IL-6 acts on hepatocytes to induce acute-phase reactants, including fibrinogen; therefore, the patients' reduced plasma fibrinogen level may have been caused by tocilizumab.

In December 2012, Matsuoka et al. reported that a 48-year-old male patient with RA developed intra-pelvic hemorrhage [18]. That patient had also received treatment with tocilizumab, and his plasma factor XIII activity level was decreased, as observed in our present case. Moreover, the factor XIII inhibitor was not detectable in the patient's blood. In our study, only the tocilizumab-treated RA patients exhibited decreased plasma factor XIII activity levels among those treated with or without biologics. The characteristic finding of tocilizumab-induced factor XIII suppression is a “slightly low” activity level (50–0%) based on the Berichrom FXIII kit. On the other hand, the factor XIII activity levels in cases of congenital factor XIII deficiency and acquired factor XIII deficiency caused by inhibitor were less than 10% and undetectable [19]–[20].

Furthermore, we measured the patients' plasma factor XIII concentrations using ELISA. There was a positive correlation between the factor XIII activity levels and plasma concentrations (r = 0.449, *P* = 0.019), and the concentrations in the tocilizumab group were lower than those observed in the TNF group. We suppose that acquired factor XIII deficiency caused by tocilizumab results from the quantitative reduction of factor XIII in the plasma. Tocilizumab possibly suppresses the production of FXIII-A and/or FXIII-B in platelets, megakaryocytes, monocytes/macrophages and/or hepatocytes.

In the multiple regression analysis, the treatment with tocilizumab was found to be an independent risk factor for plasma factor XIII reduction in the RA patients. At the same time, a male gender was also found to be an independent risk factor in the RA patients. Both our one case and the one previously reported case involved male RA patients. However, our multiple regression analysis included only eight males; therefore, further studies are needed.

Factor XIII is related to innate immune mechanisms, as well as coagulation. It has been reported that human factor XIII sequesters bacteria (for example, *Staphylococcus aureus*) in the clot matrix [21]. Factor XIII might also enhance the phagocytic activity of macrophages, because macrophages which had increased FXIII-A expression exhibited enhanced phagocytic activity for sensitized red blood cells and complement-coated yeast *ex vivo* [22]. Therefore, factor XIII might be important for bacterial killing. It has also been demonstrated that factor XIII-deficient mice develop severe pathological inflammation at local sites of streptococcal skin infection, and local factor XIII administration reduced the bacterial dissemination during the early stage of infection in wild-type animals [23]. Furthermore, monocytes obtained from factor XIII deficient mice lacked phagocytic activity. On the other hand, IL-6 is also related to the development of bacterial infections. For example, there was a case report of a child with autoantibodies against IL-6 who suffered from cellulitis and subcutaneous abscesses caused by *S. aureus* [24]. According to a Japanese post-marketing survey, approximately 12% of all infectious events in patients under tocilizumab treatment consisted of cellulitis [25], and *Staphylococcus* or *Streptococcus* species accounted for about 80% of all severe soft tissue infections acquired during treatment with tocilizumab. Nguyen, et al. reported three cases of severely disseminated *S. aureus* infection occurred in patients treated with tocilizumab [26]. Van de Sande, et al. also reported a case of necrotizing fasciitis caused by *Streptococcus pyogenes* [27]. These types of opportunistic infections had a common tendency, for example, severe skin and soft tissue infections with gram positive cocci. The skin opportunistic infections that occurred during treatment with tocilizumab might be related to the reduction of factor XIII.

In conclusion, RA patients treated with tocilizumab, an IL-6R blocker, are at risk of developing acquired factor XIII deficiency. The mechanism underlying the reduced factor XIII activity level observed in patients treated with tocilizumab may involve its quantitative reduction in the plasma. These data imply that IL-6 plays an important role in maintaining the factor XIII activity level. Our data also suggest that plasma factor XIII measurement is recommended for some RA patients, including those who have a bleeding tendency (or any symptoms caused by factor XIII deficiency) during treatment with tocilizumab, and male patients before and during treatment with tocilizumab. However, in our case, the reduction in the factor XIII activity level was mild: therefore, it does not quite explain why tocilizumab treatment alone directly caused the hemorrhage. When hemorrhagic events occur, physicians should consider discontinuing tocilizumab treatment. However, tocilizumab can provide beneficial effects for several diseases (RA, reactive AA amyloidosis, etc.). Therefore, the use of tocilizumab should not be decided based only on the plasma factor XIII activity. Further studies about the dynamics and effects of plasma factor XIII in RA patients are needed.
